# Single Injection of Bupivacaine for Correction of Strabismus

**Published:** 2015

**Authors:** Kimia Ziahosseini, Ian Bruce Marsh

**Affiliations:** Ophthalmology Department, Aintree University Hospital, Liverpool, UK

**Keywords:** Bupivacaine, Strabismus, Angle of Deviation, Esodeviation, Exodeviation

## Abstract

A retrospective review of 30 patients with eso/exodeviations less than 20 prism diopters (PD) who underwent one injection of BPX into their medial/lateral rectus was carried out. Preoperative deviations were recorded in PD, using the alternate prism cover test. Postoperative angles at 1 and 3 months were measured in the same manner. 11 out of 30 patients achieved an acceptable outcome and required no further intervention.

The mean angle of deviation for near in all patients reduced significantly by 3.1 PD and 2.1 PD at 1 and 3 months. The mean angle of deviation for distance in all patients also decreased significantly by 2.3 PD and 1.9 PD at 1 and 3 months. In conclusion, single injection of BPX in a heterogeneous group of patients with horizontal strabismus caused a mild improvement and a qualitative success rate of 37%.

## INTRODUCTION

Bupivacaine hydrochloride (BPX) is a sodium channel blocker local anesthetic that also displaces calcium from membrane binding sites and increases cytoplasmic Ca2+ level. This results in a particular myotoxixcity that leaves the basal lamina, satellite cells, nerve fibers and vessels intact allowing regeneration of muscle fibers (1, 2).

Unintentional injection of BPX into the extraocular muscles during the local anesthesia for cataract surgery can cause strabismus due to muscle hypertrophy and increased stiffness (3-5).

Scott et al showed for the first time that the injection of BPX into the lateral rectus corrected esotropia in 4 of 6 patients. The average improvement in alignment for all patients was 8 PD, with associated 6.2% increase in muscle volume measured from MRI images. Later they reported an average improvement of 19.7 PD in alignment when treated the affected muscle with BPX and its antagonist with botulinum type A toxin (6-8). Hopker et al reported an average improvement of 10 PD after six months using combination of BPX and botulinum toxin in eight patients (9). More recently, Miller et al have shown lasting changes in alignment and extraocular muscle shape after injection of BPX alone or in combination with botulinum toxin in concomitant non-paralytic strabismus (10).

 We report the outcome of a single injection of BPX for correction of small angle (<20 PD) horizontal strabismus in a heterogeneous group of patients.

## METHODS

This is a retrospective review of all patients injected with BPX at a referral center in Liverpool, UK, from March 2008 to February 2013. Eventually, this study included thirty (15 male, 15 female) patients with horizontal strabismus. Four additional patients also received BPX injection but were lost to follow- up after injection and therefore not included in this report. BPX was injected into their lateral (LR) or medial rectus (MR) muscle, depending on diagnosis. This method has been part of the standard management of patients with small angle deviation (≤ 20 PD) and in those with multiple previous surgeries who preferred a non-surgical alternative for small residual deviations.

Institutional audit approval was obtained. Marcaine (AstraZeneca, Bedfordshire, UK) - bupivacaine hydrochloride injection containing 0.5 g/dl bupivacaine, in individual dose of 4.5 to 5 ml was injected into the LR or MR under topical anesthesia (Minims® Oxybuprocaine Hydrochloride 0.4% w/v, Bausch & Lomb, Kingston-upon-Thames, UK) using a special needle insulated apart from the bevel and electromyography from the bevel. Maximal electrical signal indicated the area of greatest density of neuromuscular junctions and at that point the BPX was injected. A single surgeon (I.B.M) carried out all injections.

Preoperative measurements of the horizontal deviations of all patients were recorded in PD, using the alternate prism cover test at both 1/3 meter and 6 meters for all patients. Postoperative deviations were measured in the same manner at 1 and 3 months.

The following information were recorded as much as possible; age at presentation, best corrected visual acuity, diagnosis, detail of previous surgery and chemodenervation, sensory status, angle of deviation before and at 1 and 3 months after injection, outcome and complications. Patients with thyroid eye disease were excluded.

Values of angle of deviation are given as mean+/- standard deviation (95% confidence interval). Repeated Measures ANOVA and Wilcoxon paired test were employed to assess the comparison of the angle of deviation before or 1 and 3 months after the injection. P values less than 0.05 are considered significant. Graphpad prism 6 (Graphpad Software, Inc. La Jolla, CA, USA) was used for statistical analysis.

## RESULTS

The age distribution of our patients was between 20 and 81 years with a mean of 43.4 (median = 40). The average visual acuity was 0.1 (range: 0.2 to 1.0) LogMAR. Five patients had visual acuity worse than 0.8 LogMAR in the deviating eye.

Sixteen patients (seven females and nine males) with esotropia underwent one injection of BPX into their LR, (right: 8, left:8). Their average age was 44 (range: 24–81) years. Eight patients had previous surgery on their LR and were left with small angle residual esotropia, two had residual esotropia following recovery from abducens nerve palsy. None had chemodenervation of LR but three had previous MR chemodenervation.

**Table 1:** Comparison of the mean angle of deviations among the groups

		**Pre-op**	**1 month**	**3 months**	**Change at 1m**	**Change at 3 m**
**Esodeviation** **n=16**	Distance ANOVA P=0.008	9.6	7.1	7.5	2.5	2.1
	Near ANOVA p>0.05	9.9	7.6	8.3	2.3	1.6
**Exodeviation** **n=14**	Distance ANOVA P>0.05	7.9	5.9	6.2	2.0	1.7
	Near ANOVA p=0.06	11.3	7.1	8.6	4.2	2.7
**Combined** **n=30**	Distance ANOVA P<0.05	8.8	6.5	6.9	2.3	1.9
	Near ANOVA P<0.05	10.5	7.4	8.4	3.1	2.1

The average preoperative distance angle in LR group was 9.6 +/- 5.0 (95% CI, 6.9 to 12.3). At 1 and 3 month after injection, it reduced significantly to 7.1+/-4.7(95% CI, 4.6 to 9.6), and to 7.5+/- 4.9 (95% CI, 4.8 to 10.1) respectively, P=0.0086. The difference between the mean angle at 1 and 3 months after injection did not reach statistical significance (Table 1). The average preoperative near angle in LR group was 9.9+/- 5.2 (95% CI, 7.1 to12.7). At 1 month after injection, it reduced to 7.6+/- 4.7, (95% CI, 4.8 to 10.3) and to 8.3+/- 5.1, (95% CI, 5.4 to 11.2) at 3 months. These reductions did not reach statistical significance (Table 1).

Out of 16 patients who had local anesthetic (LA) injection into their LR for esodeviation; 5 improved to the extent that patients were content and discharged, while 11 required surgery. Among these 11 patients, five were better initially but not completely cured and therefore needed surgery while the other six did not change significantly. At 3 months, the angle of deviation improved to ≤10 PD for distance in 13 of 16(81%) and for near in 11 of 16(69%) compared to 8 of 16(50%) at pre-op for distance and near.

Fourteen patients (8 females and six males) with exotropia had one injection of BPX into their MR muscle, (right:7, left:7). Their average age was 43 (range: 20–60) years. 10/14 patients had previous surgery on their MR and were left with small angle residual exotropia. None had chemodenervation. The average preoperative distance angle in MR group was 7.9 +/- 5.1(95% CI, 4.9 to 10.9). At 1 and 3 months after injection, it reduced to 5.9+/-4.0 (95% CI, 2.8 to 8.9), and to 6.2+/- 4.7 (95% CI, 2.8 to 9.6), p>0.05(Table 1). The average preoperative near angle in MR group was 11.3+/- 4.9, (95% CI, 8.3 to 14.3). At 1 and 3 months after injection, it was reduced to 7.1+/- 5.5 (95% CI, 2.9 to 11.3) and 8.6+/- 6.8, respectively (95% CI, 4.5 to 12.7), P=0.06 (Table 1). Out of 14 patients who had LA injection into their MR, 6 were improved to the extent that patients were contented and discharged, 4 had successful surgery, 2 were lost to follow-up, 1 is managed with prism and one did not want further intervention. At 3 months, the angle of deviation improved to ≤10 PD for distance in 11 of 14(74%) compared to 10 of 14 (71%) preoperatively and for near in 8 of 14(57%) compared to 6of 14 (43%) before the injection.

The overall pre-op angle of deviation for distance was 8.8+/- 5.1 (95% CI, 6.9 to 10.7), reduced to 6.5+/- 4.4 (95% CI, 5.0 to 8.7) at 1 month and to 6.9+/-4.3(95% CI, 5.2 to 9.1) at 3 month, p=0.02(Table 1). The overall pre-op angle of deviation for near was 10.5+/- 5.1 (95% CI, 8.5 to 12.4) and reduced to 7.4+/- 4.9 (95% CI, 5.3 to 9.5), or 8.4+/-5.8(95% CI, 6.2 to 10.7) at 1 and 3 months, respectively (Table 1).

The mean improvements between eso- and exotropia groups were not significantly different. Eleven out of thirty patients achieved an acceptable angle with a single injection of BPX that no further intervention was necessary. There were no complications. There was no significant correlation between pre-op angle and the change in the angle of deviation in any group.

## DISCUSSION

Pharmacologic treatment of strabismus is an exciting and evolving field. This study represents the outcome of BPX injection in the management of a heterogeneous group of patients with small angle horizontal strabismus.

The overall reduction in the angle of deviation decreased during four months of follow-up with clinical success rate of 37%. The largest reduction occurred in the exodeviation group one month after the injection of the MR muscle. 

Miller et al have recently shown a reduction of 4.7 PD at 11.3 months after a single injection of BPX with stability of alignment changes up to 3 years (10). All their measurements were at 3 meters and therefore are not directly comparable with our results.

Earlier Scott et al showed an average reduction of 8 PD with BPX alone and 19.7 PD reduction when combined with botulinum type A toxin into the antagonist (6–8). Wutthipan et al assessed 20 patients (14 exodeviations, 6 esodeviations) at 1, 3, 6 and 12 months after BPX injection (11). Majority of their patients had an angle of deviation greater than 25PD. They noticed an improvement in 15 of 20 patients though not statistically significant. They also did not specify the distance of measurements.

Our results support others’ findings as over one-third of our patients achieved satisfactory outcome after one injection. However, the success rate and the overall improvement are more modest than in the other groups. This may be due to the selection of patients with deviations less than 20PD or due to the difference the angle of deviations were measured and reported. Namely, these measurements in our study were performed at both 1/3 and 6 meters fixation targets. Scott et al also used a higher concentration of BPX (0.75% -3%) and higher volume compared to us (7). So, other options were offered to those who failed to achieve satisfactory outcome after one injection of BPX.

It is well recognized that LR plays a more significant role in distance deviation and MR in near deviation. Therefore, we are of the opinion that simply reporting the angle of deviation for 3 meters should be avoided. When measuring small angle deviations, difficulty exists to establish whether the small changes represent a true change or are simply measurement error. Holmes et al have shown that interobserver test-retest reliability for alternate prism cover test can be as large as 10PD (12). Miller et al validated their measurement technique and showed good inter-observer reliability (10). We could not do the same due to the retrospective nature of our study.

Interestingly, Miller et al have reported on long-term stability of changes achieved (up to 3 years), although they had limited number of patients with 8, 6 and 3 patients after 1, 2 and 3 years (10).

Increase in the mean angle of deviation at 3 months compared to 1 month, did not reach statistical significance. On the other hand, none of our 11 patients that were discharged with satisfactory outcome at 3 month have come back with a recurrence after a minimum of 8 months following injection. There is of course the slim possibility that they have sought treatment elsewhere.

Since BPX is, apparently, not effective in chronically denervated muscles, Miller et al excluded paralytic strabismus from their analysis (10). We also did not find encouraging results in two patients with partial recovery from abducens nerve palsy palsy. One with left LR palsy due to multiple sclerosis with primary esodeviation of 6PD for near and 8PD for distance got better at 1 month (near 4PD, distance 2PD), but reverted to the initial angles at 3 months and underwent surgery. The other with ischemic left LR palsy and primary esotropia of 14PD for near and 18PD for distance did not change after injection and required surgery. When we excluded these patients, our results did not significantly change.

As any other retrospective study, ours is burdened with some classical limitations, such as: relatively small number of cases, which may explain the lack of statistical significance. Despite this, we believe that these results are helpful to the individual cases in clinical practice. Therefore, we believe we should continue to inform our patients of the 37% success rate when counseling.
